# Alemtuzumab induction combined with reduced maintenance immunosuppression is associated with improved outcomes after lung transplantation: A single centre experience

**DOI:** 10.1371/journal.pone.0210443

**Published:** 2019-01-15

**Authors:** Alberto Benazzo, Stefan Schwarz, Moritz Muckenhuber, Thomas Schweiger, Gabriela Muraközy, Bernhard Moser, José Matilla Sigüenza, György Lang, Shahrokh Taghavi, Walter Klepetko, Konrad Hoetzenecker, Peter Jaksch, Cristopher Lambers

**Affiliations:** Division of Thoracic Surgery, Department of Surgery, Medical University of Vienna, Vienna, Austria; Medical University of Gdansk, POLAND

## Abstract

**Question addressed by the study:**

The value of induction therapy in lung transplantation is controversial. According to the ISHLT, only about 50% of patients transplanted within the last 10 years received induction therapy. We reviewed our institutional experience to investigate the impact of induction therapy on short- and long-term outcomes.

**Materials/Patients and methods:**

Between 2007 and 2015, 446 patients with a complete follow-up were included in this retrospective analysis. Analysis comprised long-term kidney function, infectious complications, incidence of rejection and overall survival.

**Results:**

A total of 231 patients received alemtuzumab, 50 patients antithymocyte globulin (ATG) and 165 patients did not receive induction therapy (NI). The alemtuzumab group revealed the lowest rate of chronic kidney insufficiency (NI: 52.2%; ATG: 60%; alemtuzumab: 36.6%; p = 0.001). Both, the NI group (p<0.001) and the ATG group (p = 0.010) showed a significant increase of serum creatinine during follow-up compared to alemtuzumab patients. Furthermore, alemtuzumab group experienced the lowest rate of infection in the first year after transplantation. Finally, improved survival, low rates of acute cellular rejection (ACR), lymphocytic bronchiolitis (LB) and chronic lung allograft dysfunction (CLAD) were found in patients treated either with alemtuzumab or ATG.

**Conclusion:**

Alemtuzumab induction therapy followed by reduced maintenance immunosuppression is associated with a better kidney function compared to no induction and ATG. Survival rate as well as freedom from ACR and CLAD were comparable between alemtuzumab and ATG.

## Introduction

Lung transplantation remains the only therapeutic option for a variety of end-stage lung diseases, however its long-term outcome continues to be poor [1]. Calcineurin inhibitor (CNI)-based "triple-drug" maintenance immunosuppressive regimens are routinely used in lung transplantation. Nevertheless, the use of this lifelong medication results in increased risk of renal dysfunction, severe infections, malignancies and other comorbidities such as arterial hypertension, osteoporosis and neurological disorders and thereby resulting in reduced quality of life and long-term survival [2–4]. According to the 33rd report from the Registry of the ISHLT [1], only 50% of patients transplanted within the past 10 years received some type of induction therapy. The rationale behind its use is to prevent acute cellular rejection (ACR) episodes, to delay initiation of maintenance immunosuppression and to reduce of its cumulative dose.

Currently the majority of lung transplant centres use IL2R Antagonists (IL2RA) reporting varying outcomes after lung transplantation [5–12]. Polyclonal anti-lymphocyte preparations (ATG) are the second most used induction agents. Although they can effectively reduce ACR rates they are associated with adverse effects such as thrombocytopenia, leukopenia and anemia.

More recently, an increasing number of centers have started using alemtuzumab, a humanized monoclonal antibody targeting CD52. The receptor recognized by the antibody is expressed on several immune cells (T and B lymphocytes, Natural Killer (NK) cells and macrophages) and its activation induces cell lysis leading to immune cell depletion [13–14]. Recovery of B cells occurs between 3 to 6 months and recovery of T cells between 12 to 24 months [13, 15]. After reconstitution, T cells show an increased fraction of CD4^+^CD25^high^ T cells whereas recovered B cells consist mainly of IgM-producing näive B cells and transient B cells [13, 15]. In stable immunosuppression-free kidney recipients, a higher percentage of the same subset of B cells was found, thus proposing the hypothesis that such phenotypic changes in B cells play a crucial role in allograft tolerance mechanisms [16]. Based on these findings it is tempting to speculate whether alemtuzumab induction therapy could create an environment immunological tolerance. In 2014, our group published the first prospective randomized trial comparing Alemtuzumab and ATG as induction therapy. Thirty patients were included in each group and followed for approximately two years. T and B cells were depleted in both groups, however patients receiving alemtuzumab experienced stronger long-lasting lymphocyte depletion. Although complete absence of ACR was observed in patients treated with alemtuzumab in the first year after transplantation, the two groups did not significantly differ in terms of survival, freedom from CLAD and kidney function [17].

In the current study we retrospectively reviewed all adult lung transplant recipients operated between 2007 and 2015. Patients were stratified according to the use of induction therapy regimen chosen. Analysis comprised patient survival, CLAD development, incidence of ACR and lymphocytic bronchiolitis (LB) as well as kidney function.

### Patients and methods

Between January 2007 and January 2015, 830 adult recipients were transplanted at our centre. To guarantee uniformity of follow-up as well as data quality and data completeness, only patients who were followed up by our transplant centre were included in the study. 384 patients, who were followed up by one of our referring centres, were excluded from the analysis.

Patients received either induction immunosuppression therapy or no induction according to our clinical protocol in use and they were therefore divided into three groups: alemtuzumab (Genzyme/Sanofi), rabbit antithymocyte globulin (ATG-Fresenius S; Biotech), or no induction. Until 2009 we used ATG induction in CF and IPH patients, all other recipients received no induction. In 2009 and 2010 a randomized prospective study was performed using ATG vs Alemtuzumab irrespective of the underlying disease. Due to the encouraging results of this study Alemtuzumab was implemented as standard treatment for all recipients with exception of patients with GvHD after bone marrow transplantation and patients colonized by species of *Burkholderia* or resistant *Mycobacterium abscessus* prior to lung transplantation, who did not received any induction therapy. Prior to the implementation of alemtuzumab, severe intraoperative bleeding or the implementation of an extracorporeal life support (ECLS) device as bridge to lung transplantation represented a contraindication for the use of ATG, in order to minimize bleeding risk in the post-operative period. Since alemtuzumab is not associated with thrombocytopenia, it could also be used for patients at a higher risk for postoperative bleeding.

ATG was intravenously administrated at a dose of 2 mg/kg on postoperative day (POD) 0,1,2,3 and 4, while alemtuzumab was given as a single dose of 30 mg after arrival on the intensive care unit (ICU).

Maintenance immunosuppression regimen was based on triple-drug treatment combination of tacrolimus, mycophenolate mofetil (MMF) and steroids. Tacrolimus was started immediately after transplantation with target blood levels depending on the type of induction therapy. Target blood levels are described in Table 1. During long-term follow-up, in case of deterioration of kidney function, history of infection or rejection the target blood levels were adapted to the low or high end of the target range accordingly. MMF was started based on leukocyte count and clinical course, with a standard dose of 2-3g/day. In patients receiving alemtuzumab, MMF was usually started one year after transplantation. To lower nephrotoxicity in the presence of reduced glomerular filtration rate (GFR) (<50ml/min), patients were switched to everolimus in combination with reduced dose of calcineurin inhibitors.

**Table 1:** Maintenance immunosuppression protocol.

	Calcineurin Inhibitors				Aprednisolone mg/kg		Anti-proliferative	
	No induction /ATG		Alemtuzumab		No induction /ATG	Alemtuzumab	No induction /ATG	Alemtuzumab
	CyA ng/ml	Tacrolimus ng/ml	CyA ng/ml	Tacrolimus ng/ml			MMF	
0–3 months	300–350	15–18	200	10–12	0.3	0.2	1–1.5g twice a day	-
3–6 months		13–15		8–10	0.2	0.15	1–1.5g twice a day	-
6–12 months	250–300	10–12	150	6–8	0.15	0.1	1–1.5g twice a day	-
12–24 months	200	8–10	150	5–7	5 mg/d	5 mg/d	1–1.5g twice a day	1–1.5gtwice a day
>24 months	100–200	8	100–150	5			1–1.5g twice a day	

Perioperative infectious prophylaxis was similar in all groups and based on broad-spectrum antibiotics (piperacillin-tazobactam or meropenem) or adapted to pre-transplant resistance testing. Cystic fibrosis patients and patients with recurrent infections received antibiotics according to resistance testing. All patients received a lifelong *Pneumocystis* prophylaxis with trimethoprim-sulfamethoxazole. Prophylactic inhalation therapy with amphotericin B and gentamycin was provided for 1–3 months. CMV prophylaxis included CMV hyperimmunoglobulines (POD 1,7,14 and 21) together with valganciclovir for a minimum of 3 months. In high-risk patients (donor CMV IgG positive, recipient CMV IgG negative) a 12 months prophylaxis was performed. Intravenous or oral antifungal therapy (voriconazole or other echonicandines) was administered in patients requiring perioperative ECMO support, patients treated for ACR or in case of positive cultures.

All patients followed our standard post-transplant care protocol, including follow-up visits (once a month during the first year and later every three months), bronchoscopy with transbronchial biopsy (TBB) and bronchoalveolar lavage (BAL) (1, 2, 3, 6, 12 months after transplantation), spirometry, computed tomography (CT) scan (each year) and blood examination. Additional diagnostic TBB, BAL and CT scans were performed in case of lung function deterioration, e.g. in case of suspected acute rejection episode or infections. Biopsies were classified according to ISHLT criteria [18]. ACR grade A2 and LB B2 or higher were primarily treated with steroids (500–1000 mg/day) for 3 days with consecutive dose tapering and in case of non-response to steroids, ATG (2 mg/kg) was administered for 5 days followed by extracorporeal photopheresis if necessary. Rejection treatment always included antibiotic prophylaxis with broad-spectrum antibiotics and valganciclovir treatment. All patients with a decrease in lung function received azithromycin irrespective of induction therapy, after exclusion of treatable causes of lung allograft dysfunction (e.g. chronic infection, GERD).

The three groups (no induction, alemtuzumab, ATG) were analyzed according to the following outcome parameters: patient survival, CLAD incidence (defined according to ISHLT/ATS/ERS criteria [18]), cumulative A and B grade, high grade ACR and LB (defined respectively as ≥ A2 and B2), long-term kidney function and incidence of malignancies.

Kidney insufficiency was defined as GFR <60 mL/min/1.73 m^2^ for ≥ 3 months. Estimated GFR was calculated with MDRD formula. Infectious complications, including *Aspergillus* infection, were defined as presence of pathogens with clinical signs and symptoms and need for specific treatment. CMV outcomes were defined according to the last report of the CMV Drug Development Forum [19]. In brief, CMV disease was defined as CMV DNAemia in combination with symptoms and signs of organ involvement together with biopsy confirmed CMV infection or other appropriate specimen.

This study has been approved by the Institutional Ethical Committee of the Medical University of Vienna [ECS 1891/2016] and was conducted according the declaration of Helsinki.

### Statistical analysis

Categorical variables are reported as count and percentage and were analyzed by Chi-squared or Fischer’s exact test. Continuous variables were presented as mean ± SD or median (min-max) and compared by Kruskal Wallis H test, Mann-Whitney U-test or Kolmogorov-Smirnov Z test. Curves for survival, CLAD, ACR and LB incidence were generated with the Kaplan-Meier method and were compared with log-rank test. Univariate and multivariate cox regression was performed to find predictors of mortality, CLAD onset, ACR and LB incidence. Variables were included in the multivariable Cox regression model when they had significant associations on univariate analysis (p < 0.05) or according to clinical plausibility (for survival: age, year of transplantation, LAS score and induction therapy; for CLAD: sex, LAS score, year of transplantation, induction therapy; for ACR: year of transplantation, LAS score and induction therapy; for LB: age, year of transplantation, LAS score, pre-transplant intubation and induction therapy). Cox proportional hazard models were controlled using Kaplan-Meier plots and testing correlation coefficients of the partial residuals with time. In order to understand if induction therapy exerts an influence on long-term creatinine blood level, we built a linear mixed model including 1/creatinine prior to transplantation and at follow-up date after 3 and 6 months, 1 and 2 years, 3 years and 4 years [20]. Before running our model data were explored graphically using box plots and curves depicting creatinine versus time (individual and grouped). Covariance structure was chosen following the Akaike information criterion (AIC) yielding the smallest AIC. We adjusted the model for baseline creatinine level (pre-transplant Creatinine), including it in the covariates and we checked for time-dependent effects. Data were analyzed using SPSS version 23.0 software (SPSS Inc. Chicago. IL).

## Results

A total of 446 patients were included in this study. Fifty-two percent (n = 231) received alemtuzumab induction therapy, 11% (n = 50) ATG and 37% (n = 165) had no induction therapy at all. Median follow-up time was 1074 days (range: 1–3280)

### Population characteristics

Demographics are depicted in Table 2. ATG was used more frequently in CF and idiopathic pulmonary hypertension (iPAH) patients (p<0.001), whereas a significant number of patients with fibrotic lung diseases did not receive induction (p<0.001). In patients bridged to transplantation with mechanical ventilation or extracorporeal life support, induction therapy was used less frequently (p<0.0001). Patients without induction therapy had a higher rate of re-intubation (p<0.01), more frequently required tracheostomy in the early postoperative period (p<0.01) and had a longer ICU stay (p<0.05).

**Table 2:** Patients demographics and perioperative characteristics.

		Induction therapy			*p-value*
		No	ATG	Alemtuzumab	
Sex	Male	94 (57%)	19 (38%)	123 (53.2%)	0.062
	Female	71 (43%)	31 (62%)	108 (46.8%)	
Age		52 (21–72)	49 (19–65)	53 (19–67)	0.530
Diagnosis	COPD	76 (46.1%)	25 (50%)	117 (50.6%)	**<0.001**
	Fibrosis	51 (30.9%)	2 (4%)	37 (16%)	
	iPAH	8 (4.8%)	6 (12%)	16 (6.9%)	
	CF	11 (6.7%)	16 (32%)	48 (20.8%)	
	Others	19 (11.5%)	1 (2%)	13 (5.6%)	
Type of Tx	DLuTX	153 (92.7%)	47 (94%)	226 (97.8%)	0.083
	SLuTX R	4 (2.4%)	0 (0%)	1 (0.4%)	
	SLuTX L	8 (4.8%)	3 (6%)	4 (1.7%)	
CMV risk	D+/R-	27 (17%)	9 (18%)	48 (21.4%)	0.110
	D+/R+	78 (49.1%)	26 (52%)	93 (41.5%)	
	D-/R+	42 (26.4%)	6 (12%)	55 (24.6%)	
	D-/D-	12 (7.5%)	9 (18%)	28 (12.5%)	
Pre-Tx intubation		29 (17.6%)	3 (6%)	8 (3.5%)	**<0.001**
Weaning from invasive ventilation (days)		2 (0–63)	1 (1–25)	2 (1–210)	0.060
ICU time (days)		10 (1–124)	7 (1–105)	7 (1–216)	**0.032**
Hospitalization time (days)		25 (1–160)	23 (8–105)	22 (1–246)	0.128
ECLS Bridging to Tx		21 (12.7%)	0 (0%)	9 (3.9%)	**<0.001**
Postoperative prolonged ECMO		44 (26.7%)	7 (14%)	49 (21.2%)	0.139
In-hospital death		21 (12.8%)	4 (8.2%)	14 (6.1%)	0.065
Post-Tx Tracheostomy		61 (37%)	14 (28%)	48 (20.8%)	**0.002**
Revision surgery		30 (18.2%)	7 (14%)	37 (16%)	0.741

### Kidney function

Pre-transplant kidney function was comparable in all groups (Table 3).

**Table 3:** Patients comorbidities during long-term follow-up.

		Induction therapy			*p-value*
		No	ATG	Alemtuzumab	
Pre-Tx kidney insufficiency		12 (7.4%)	2 (4%)	6 (2.6%)	0.810
Post-Tx kidney insufficiency		84 (52.2%)	30 (60%)	83 (36.6%)	**0.001**
Pre-Tx diabetes		22 (14%)	9 (18.8%)	38 (16.7%)	0.668
Post-Tx diabetes		28 (19.7%)	16 (35.6%)	60 (27.1%)	0.740
First Infection	< 3 months	47 (35.3%)	17 (36.2%)	47 (21.4%)	**0.001**
	3-6months	29 (21.8%)	8 (17%)	39 (17.7%)	
	6–12 months	27 (20.3%)	10 (21.3%)	35 (15.9%)	
	>1 year	21 (15.8%)	9 (19.1%)	50 (22.7%)	
Pseudomonas colonization	First year	25 (17.5%)	14 (29.8%)	29 (13.2%)	**<0.001**
	Permanent	14 (9.8%)	9 (19.1%)	25 (11.4%)	
Aspergillus infection		10 (6.3%)	7 (14.3%)	20 (8.8%)	0.204
CMV disease		11 (7.1%)	3 (11.5%)	12 (5.3%)	0.766
Malignancy	No	159 (96.4%)	47 (95.9%)	224 (97%)	0.685
	PTLD	3 (1.8%)	2 (4.1%)	5 (2.2%)	
	Solid tumors	3 (1.8%)	0 (0%)	2 (0.9%)	
Cumulative A score		1.11±1.3	0.73±1.27	0.30±0.77	**<0.001**
Cumulative B score		3.06±2.41	3.03±2.36	1.40±1.89	**<0.001**

Of note, patients receiving Alemtuzumab had a lower incidence of kidney insufficiency (GFR <60ml/min) during their follow-up (no induction: 52.2%; ATG: 60%; Alemtuzumab: 36.6%; p = 0.001). Prior to transplantation, patients in the three groups had comparable creatinine serum levels, however, during the first three post-transplant years the alemtuzumab group maintained significantly lower serum creatinine levels (Table 4).

**Table 4:** Median serum creatinine level during long-term follow-up.

	Induction therapy			*p-value*
	No	ATG	Alemtuzumab	
Before Tx	0.8 (0.15–8)	0.79 (0.42–1.49)	0.78 (0.23–3.2)	0.102
3 months	1.22 (0.44–3.79)	1.1 (0.54–2.1)	1.07 (0.25–3.75)	**<0.001**
6 months	1.42 (0.62–6.32)	1.28 (0.7–2.7)	1.22 (0.3–3.1)	**<0.001**
1 year	1.52 (0.63–5.74)	1.48 (0.56–3.2)	1.28 (0.47–4.2)	**<0.001**
2 years	1.48 (0.7–5.27)	1.48 (0.8–3)	1.24 (0.63–3.36)	**<0.001**
3 years	1.56 (0.7–7.38)	1.45 (0.92–4.9)	1.31 (0.37–3.05)	**0.015**
≥ 4 years	1.46 (0.6–7.65)	1.37 (0.85–6.89)	1.30 (0.80–3.97)	0.875

**Fig 1:**
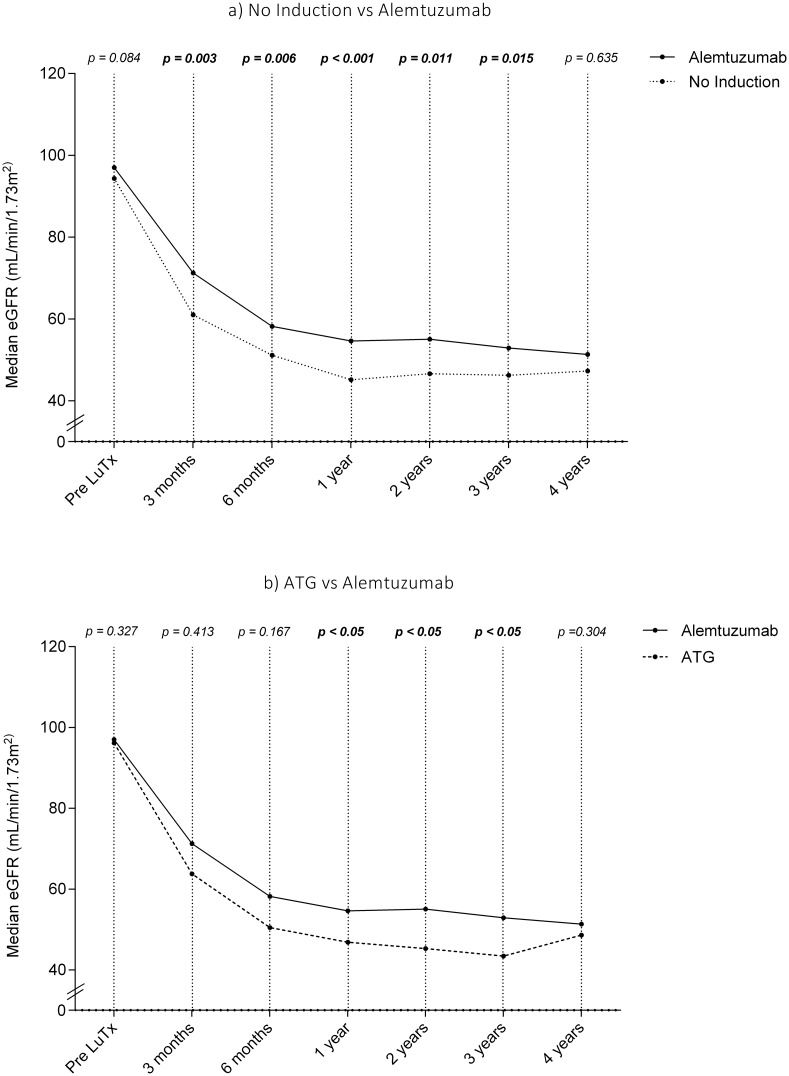
Trend of estimated glomerular filtration rate after lung transplantation overtime: a) comparison between NI and alemtuzumab, b) comparison between ATG and alemtuzumab.

Similar results were obtained when eGFR was calculated using the Modification of Diet in Renal Disease (MDRD) formula (Fig 1).

Median eGFR were comparable in all three groups prior to transplantation. Naturally, all three groups showed a decrease of eGFR following transplantation, however, patients in the alemtuzumab group had a significantly higher eGFR at all time points within the first three years post-transplant compared to NI and ATG patients (Table 5).

**Table 5:** Median eGFR during long-term follow-up.

	Induction therapy			*p-value*
	No	ATG	Alemtuzumab	
Before Tx	94.14 (5.4–534.9)	96.1 (43.9–223.9)	100 (23.2–552)	*0.084,**0.327
3 months	61 (13.24–221)	63.74 (25.5–133.8)	71.4 (18.2–365)	*0.003, **0.413
6 months	51.13 (10.6–128.6)	50.5 (21.8–114.6)	58.6 (17.8–244.6)	*0.006, **0.167
1 year	45.1 (7.97–114.38)	46.9 (18.1–125)	56.2 (15.8–167.7)	*<0.001,**<0.05
2 years	46.6 (9.6–126.2)	45.3 (16.9–94.6)	56.4 (15.5–156.3)	*0.011, **<0.05
3 years	46.2 (5.93–110.19)	43.4 (14.1–85)	57 (21.8–221)	*0.015, **<**0.05**
≥ 4 years	47.25 (5.7–123.1)	46.03 (8.7–86.6)	50.9 (12.7–142.8)	*0.635, **0.304

* NI vs Alemtuzumab;

** ATG vs Alemtuzumab

The effect size of increase in creatinine during the first four years was calculated by a linear mixed model and it was 0.107 (CI = 0.188–0.026) (p = 0.010) in the ATG group and 0.126 (CI = 0.180–0.071) (p<0.001) in the NI group.

### Infections and malignancies

Within the first year after transplantation the incidence of infections was lowest in the alemtuzumab group (p<0.001). However, this finding was reversed beyond one year (NI: 15.8%; ATG: 19.1%; alemtuzumab: 22.7%; p<0.001). There was no significant difference between the three groups regarding incidence of aspergillus infection, CMV disease and malignancies (5 post-transplant lympho-proliferative diseases, 10 solid tumors).

### Immunosuppression

Overtime leukocyte counts are presented in Table 6. Alemtuzumab allowed a significant reduction of dosage and blood levels of tacrolimus or cyclosporine A (p<0.001) (Table 7). In addition. per protocol MMF was started immediately after transplantation in NI or ATG group but could be postponed to one year after in the alemtuzumab group.

**Table 6:** Median blood leukocyte level during long-term follow-up.

	Induction therapy			*p-value*
	No	ATG	Alemtuzumab	
3 months	10.23 (0.13–92.67)	8.87 (1.23–57.89)	8.58 (0.00–62.56)	**<0.001**
6 months	7.08 (0.09–32.61)	5.42 (0.62–17.02)	5.15 (0.06–22.97)	**<0.001**
1 year	6.77 (0.93–32.39)	4.61 (0.40–24.89)	5.405 (0.24–33.65)	**<0.001**
2 years	6.84 (0.16–31.71)	5.89 (0.34–28.19)	5.92 (0.50–35.39)	**<0.001**
3 years	7.72 (0.20–30.16)	6.8 (2.87–21.64)	6.65 (1.19–34.77)	**<0.001**
≥ 4 years	8.27 (0.18–36.16)	6.83 (0.03–21.16)	6.68 (2.44–35.36)	**<0.001**

**Table 7:** Median blood CNI (tacrolimus and cyclosporine A) levels during long-term follow-up.

	Induction therapy			*p-value*
	No	ATG	Alemtuzumab	
3 months	12.7 (2.10–120.40)	12.80 (2–86)	10.30 (2–81)	*<0.001, **<**0.001**
6 months	10.8 (2–46.7)	11.7 (2–55.6)	9.3 (2.4–55)	*<0.001, **<**0.001**
1 year	9.1 (2–54.1)	10.1 (2.7–59.1)	7.8 (2.1–48)	*<0.001, **<0.001
2 years	6.4 (2–39.6)	7.1 (2.1–22.6)	6.4 (2–34.3)	*<0.001, **<0.001
3 years	6.3 (2–41.3)	5.1 (2.1–59)	6.6 (2.1–29.7)	*0.107, **<0.001
≥ 4 years	6.2 (2.1–22.4)	4.3 (2–15.9)	5.9 (2–22.1)	*0.087, **<0.001**
	Induction therapy			*p-value*
	No	ATG	Ca	
3 months	270 (34–3298)	242 (61–738)	158 (34–1380)	*<0.001, **<0.001
6 months	213 (37–1243)	256 (31–454)	191.5 (153–343)	*0.219, **<0.001
1 year	190 (60–1984)	130 (67–486)	192 (114–257)	*0.078, **<0.001
2 years	164 (30–836)	135 (40–337)	152 (120–303)	*0.040, **<0.001
3 years	112 (31–658)	75.5 (39–289)	94 (30–443)	*0.058, **<0.05
≥ 4 years	98 (30–494)	69 (30–686)	93 (44–168)	*0.087, **<0.05

* NI vs Alemtuzumab;

** ATG vs Alemtuzumab

### Rejection

**Fig 2:**
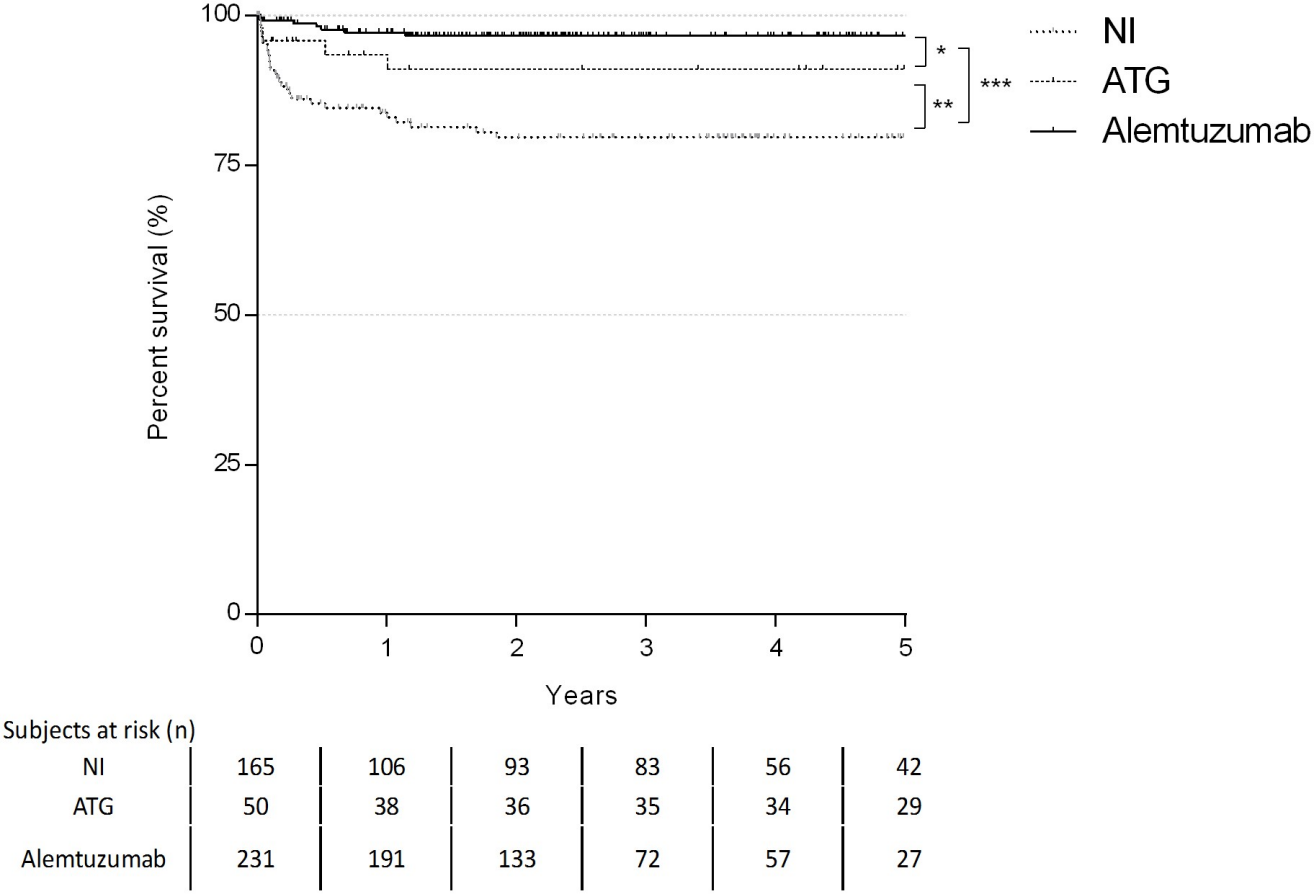
Kaplan-Maier curve for incidence of ACR (rank test: * p = 0.090; **p = 0.084; ***p<0.001).

One- and five-year freedom rates from higher grade ACR were 83% and 79.7% for NI, 91% and 91% for ATG and 97.2% and 96.7% for alemtuzumab, respectively. Use of alemtuzumab resulted in the greatest freedom from higher grade ACR (p<0.001). No statistical difference was found between the one- and five-year freedom rates of the NI and ATG group (p = 0.084) as well as between alemtuzumab and ATG group (p = 0.090) (Fig 2). In addition, alemtuzumab group showed the lowest cumulative A score during the follow-up (p<0.001) (Table 3).

**Fig 3:**
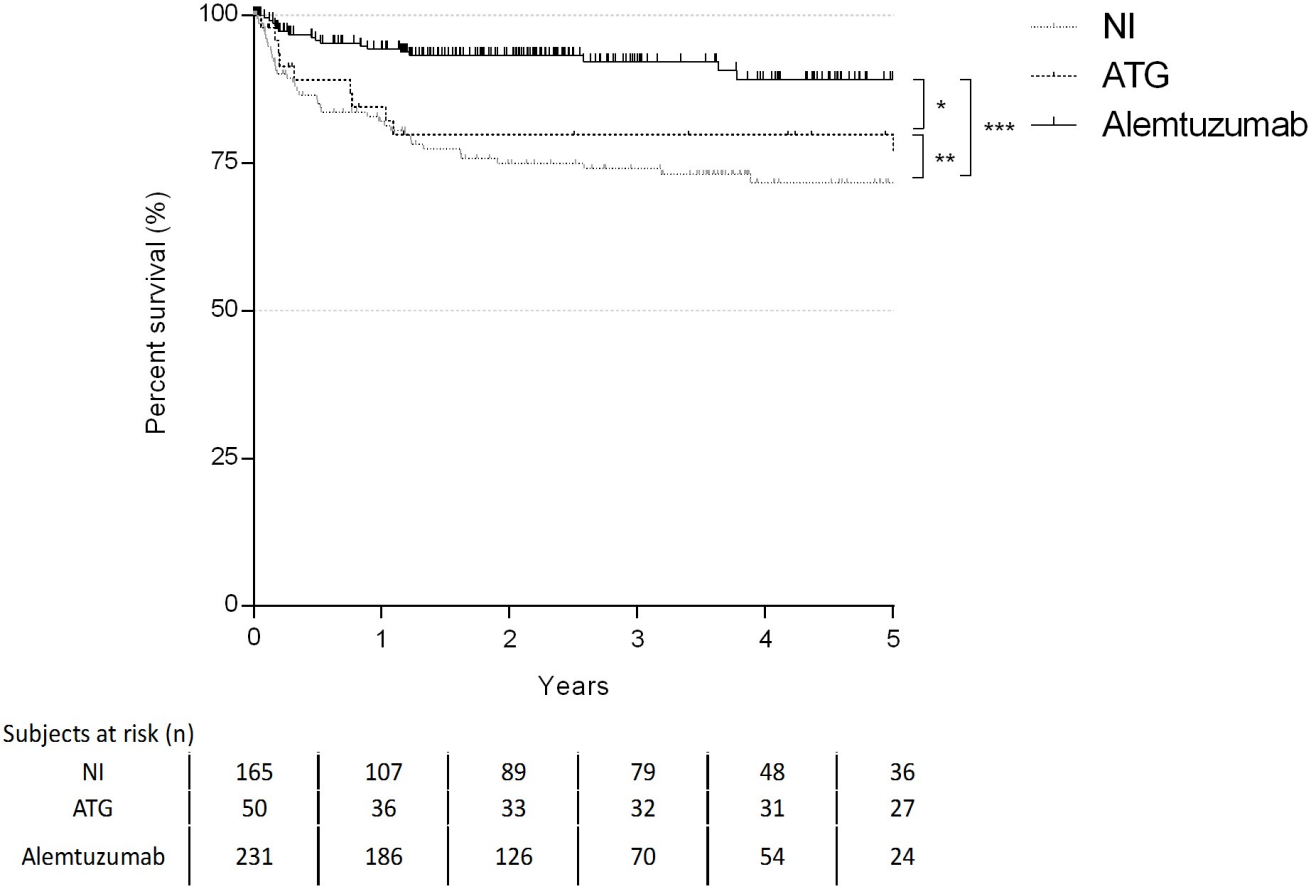
Kaplan-Maier curve for incidence of LB (log-rank test: * p<0.01;**p = 0.437;***p<0.001).

One- and five-year freedom rates of LB were 82.1% and 71.7% for NI, 84.5% and 76.9% for ATG, 94.4% and 89.2% for alemtuzumab (Fig 3). Use of alemtuzumab resulted in lowest LB grade (p<0.001). No significant differences were found between ATG and NI groups. Moreover, the alemtuzumab group showed the lowest cumulative B score during the follow-up (p<0.001) (Table 3).

**Fig 4:**
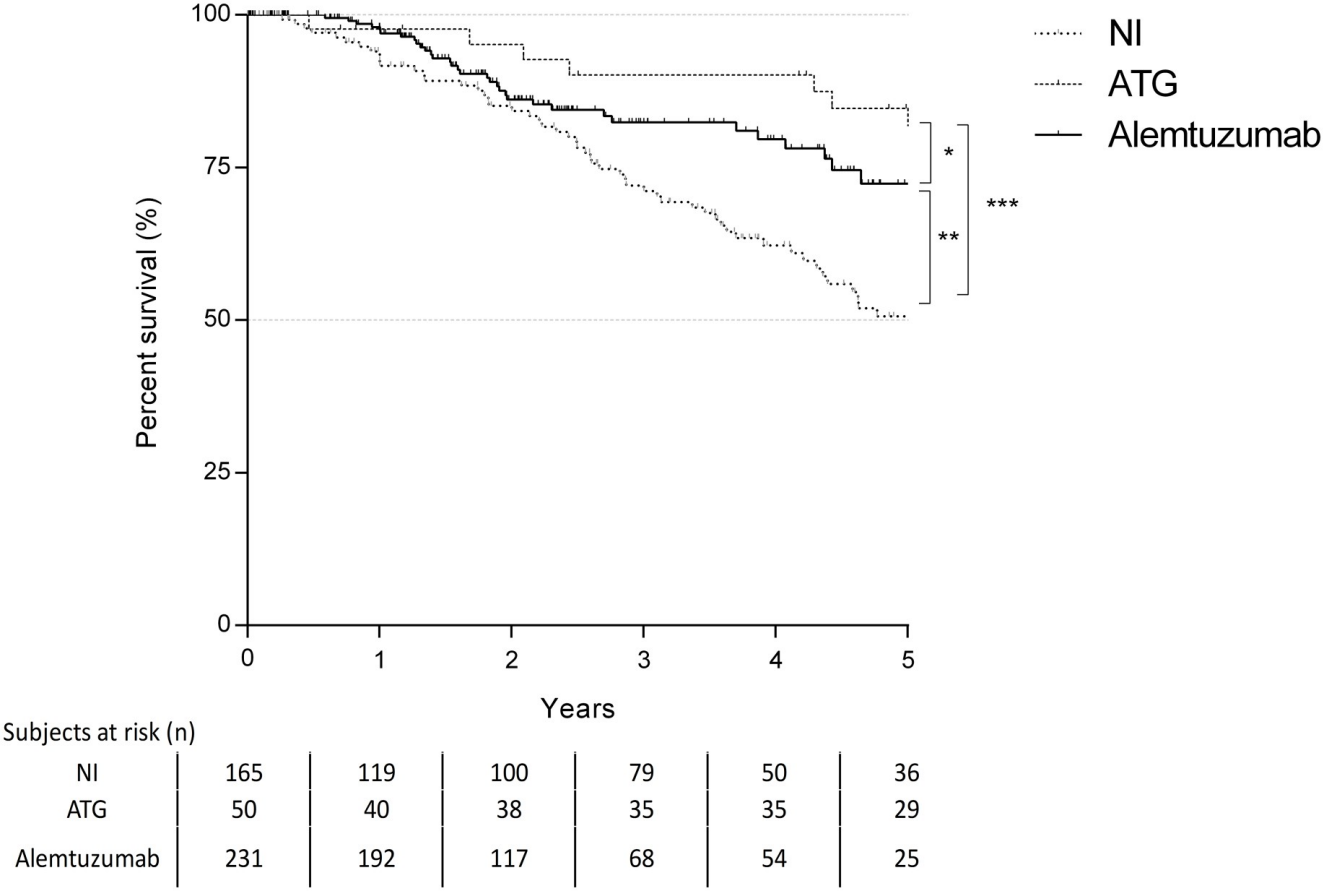
Kaplan-Maier curve for incidence of CLAD (log-rank test *p = 0,286;**p<0,001; ***p = 0,001).

Finally, 1-. 3- and 5-years freedom rates from CLAD were 92.4%, 74.8% and 50.6% for NI group, 97.7%, 90.2% and 84.7% for ATG group, 98%, 82.4% and 72.4% for alemtuzumab group (Fig 4).

Both, the ATG (p<0.01) and the alemtuzumab (p = 0.001) group were superior in terms of freedom from CLAD compared to the NI group. Eleven patients out of the entire cohort of 446 patients required retransplantation for CLAD. There was no difference between the groups in regard to diagnosis (p = 0.472). The most frequent indication for retransplantation was Bronchiolitis Obliterans (n = 7, 63.6%).

### Survival

**Fig 5:**
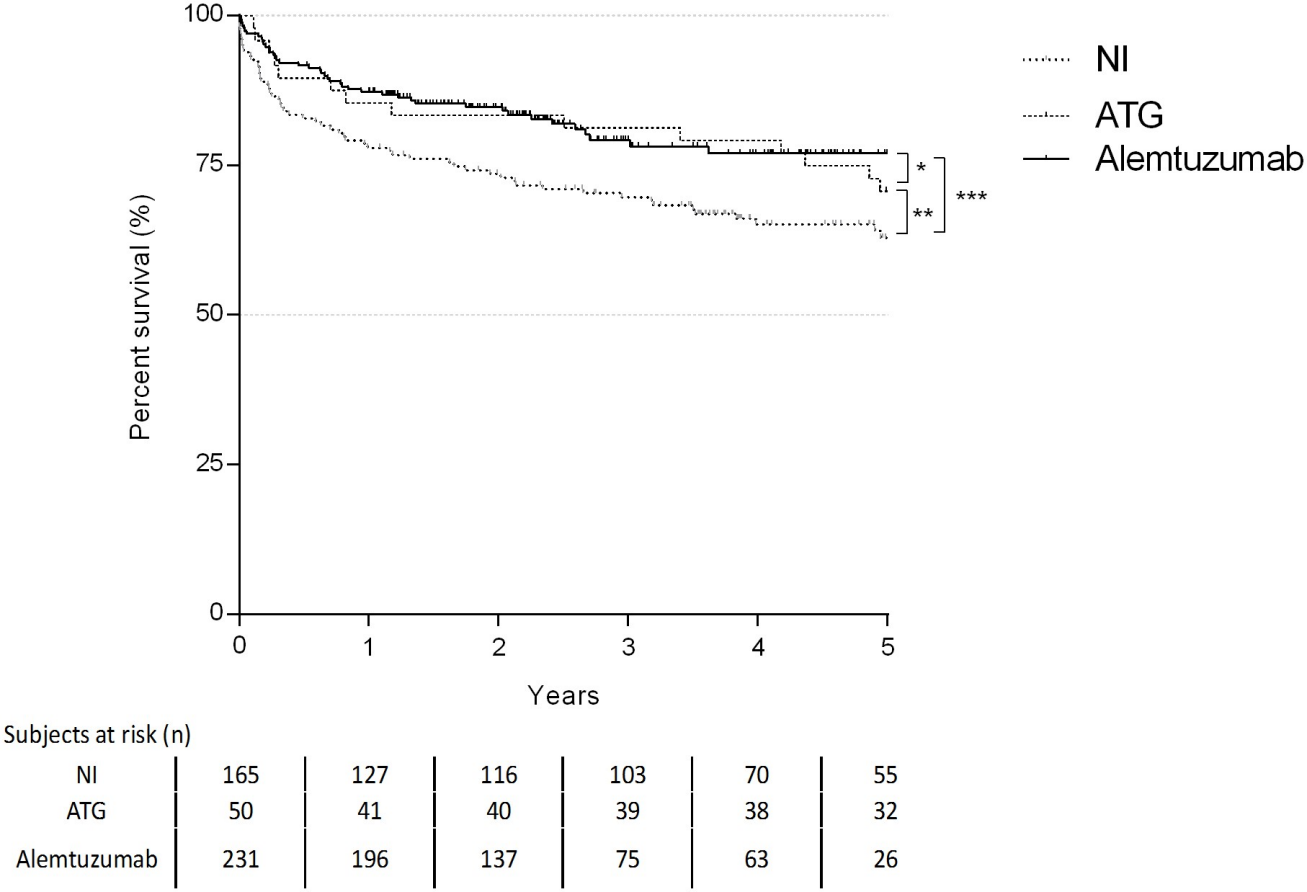
Kaplan-Maier curve for patient survival (log-rank test: * p = 0,719; **p = 0,037, ***p = 0,002).

Patients receiving alemtuzumab and ATG revealed the best survival according to the Kaplan-Meier analysis. One- and 5-years survival rates were 77.9% and 62.9% for the NI group, 85.4% and 70.7% for the ATG group and 87.3% and 77% for the alemtuzumab group (Fig 5).

Detailed causes of death for all patients are depicted in Table 8. A total of 114 patients died within the analyzed period, with the main cause being infectious complications (n = 63, 53.8%).

**Table 8:** Causes of death.

		Induction therapy		
		No	ATG	Alemtuzumab
Cause of death	Graft failure	1 (1.6%)	0 (0.0%)	1 (2.4%)
	BO	9 (14.8%)	1 (6.7%)	3 (7.3%)
	Infection-not-CMV	27 (44.3%)	10 (66.7%)	26 (63.4%)
	CMV	1 (1.6%)	0 (0.0%)	0 (0.0%)
	Cardiovascular	6 (9.8%)	0 (0.0%)	4 (9.8%)
	Others	10 (16.4%)	2 (13.3%)	2 (4.9%)
	Acute Rejection	2 (3.3%)	0 (0.0%)	2 (4.9%)
	Skin and solid malignancies	4 (6.6%)	1 (6.7%)	1 (2.4%)
	PTLD	0 (0.0%)	0 (0.0%)	1 (2.4%)

### Multivariable analysis

After testing independent covariates in univariate analysis (S3–S6 Tables), multivariable Cox regression models were constructed for survival, incidence of CLAD, ACR and LB. Variables with a significant association in univariate analysis and those with a given clinical plausibility were included in the multivariable analysis. Analysis revealed that the use of alemtuzumab and ATG were independent prognostic factors for survival when compared to NI treatment with an HR = 0.600 (CI = 0.374–0.961; p = 0.033) and HR = 0.464 (CI = 0.252–0.854; p = 0.014), respectively. For incidence of CLAD, alemtuzumab and ATG were protective factors compared to NI (alemtuzumab: HR = 0.459 CI = 0.280–0.751; p = 0.002; ATG: HR = 0.351. CI = 0.171–0.722. p = 0.004). Patients receiving alemtuzumab were less likely to develop ACR or LB compared to NI (A2: HR = 0.168. CI = 0.060–0.474. p<0.001; B2: HR = 0.480. CI = 0.243–0.948. p = 0.035). All results are depicted in S1 and S2 Tables.

## Discussion

This retrospective analysis revealed a significant survival benefit and a lower incidence of CLAD and ACR for patients receiving alemtuzumab. The use of this novel agent allowed a significant reduction of CNI cumulative dose, which lead to a better long-term kidney function compared to the other induction strategies.

The use of induction immunosuppression therapies is still controversially discussed. Despite this, an increasing numbers of leading lung transplant centers implemented induction treatment strategies in their routine [1]. Despite the promising data on the use of alemtuzumab as induction therapy, there are currently only a handful of centers using this induction agent. One of the reasons for the reluctance to use alemtuzumab are the dreaded side effects, namely severe infections and malignancies. In our analysis, an opposite effect on infections was observed. In the first year the number of severe infections was, in fact, significantly lower compared to ATG and the NI group. Fifteen patients developed malignancies but the type of induction therapy was not associated with their occurrence (p = 0.685. Table 3).

To date, published data on the use of alemtuzumab in lung transplantation are scarce. Van Loenhout et al. retrospectively analyzed outcome of 20 patients receiving alemtuzumab induction therapy. Their analysis revealed no significant difference between the active group and the control group regarding survival, ACR and LB incidence and infections. They observed a lower blood creatinine and glucose level in association with a lower level of maintenance immunosuppression [21]. However, this retrospective study included only a small number of patients, followed for 1 year after transplantation [21]. In a subsequent analysis published in 2011, Shyu and colleagues reviewed their 5-year experience with alemtuzumab induction. The alemtuzumab group evidenced improved patient and graft survival compared to the non-induction and daclizumab group. Moreover, freedom from ACR, lymphocytic bronchiolitis (LB) and bronchiolitis obliterans (BO) was greater [22] in the alemtuzumab group. CLAD incidence was lower and comparable to the group receiving Thymoglobulin. A similar retrospective analysis [23] of 89 patients (44 basiliximab, 45 alemtuzumab) showed a lower average biopsy score and a lower incidence of higher grade ACR in alemtuzumab-treated patients. Furthermore, the incidence of infections was comparable between the groups. The authors could not find a difference in creatinine blood level, however, according to their protocol the level of tacrolimus was not reduced in alemtuzumab group and patients were only followed 6 months [23]. Furuya and colleagues, reviewed the UNOS database for double lung transplant recipients and identified 738 patients, who had received alemtuzumab induction therapy [24]. These patients had a significant better survival and showed a lower risk for CLAD. Finally, our center conducted the only prospective randomized controlled trial published to date comparing alemtuzumab and ATG. In this study, a significant reduction of higher grade rejection rates with alemtuzumab induction followed by reduced doses of tacrolimus was demonstrated [17]. Based on the so far published experience, induction therapy with alemtuzumab and ATG seem to be associated with a better survival and a significantly reduced incidence of CLAD. The findings presented in the current study are in line with the published data in the medical literature.

As presented in Table 2, demographical data of the three groups differed in relation to diagnosis and pre-transplant status (i.e. pre-transplant intubation or ECMO bridging to transplantation). These differences can be attributed to the retrospective nature of the study. In addition, the selection of the induction therapy was also dependent on the chronological availability of the induction agent rather than on pre-transplant general status of the candidate. In the study period an increasing number of CF patients underwent transplantation while ATG and alemtuzumab were implemented into our immunosuppressive protocol. Cystic fibrosis patients with a pre-transplant colonization of *Burkholderia* or *Mycobacterium abscessus* (n = 13, 2.9%), moreover, did not receive alemtuzumab, thus attempting to reduce the risk of reactivation and infectious complications. As mentioned above the agent induces stronger and longer lymphocyte depletion after transplantation, which can last up to 24 months.

Patients bridged to transplantation are more prevalent in the NI group. Prior to alemtuzumab, patients on ECLS, did not receive ATG. Both, ECLS and ATG, can cause severe thrombocytopenia and increase the perioperative risk for bleeding when combining both therapies was considered too high. Only in the last years, bridge to transplantation evolved in the awake bridging concept and concomitantly alemtuzumab was implemented in our immunosuppression strategy. Accordingly, since 2010 the number of intubated patients receiving a transplantation has been reduced from 8 to 2 per year. To clarify the role of diagnosis, bridging strategies (mechanical ventilation vs. ECLS) and the role of an era effect on patient and graft survival, we performed a multivariable cox regression, however no significant associations were found.

The potential benefit of induction therapy is two-fold: (i) to reduce the likelihood of early acute rejection episodes and (ii) to decrease the cumulative burden of immunosuppressive (IS) medication. ATG had the desired effect of reducing the number of acute rejection episodes, which most likely led to a decrease in chronic lung allograft dysfunction [25–27]. However, ATG did not allow a clinically meaningful reduction of maintenance IS. Therefore, general side effects of CNI-based IS regimen remained the same. The main advantage of alemtuzumab in our patient cohort was the possibility to reduce maintenance IS dosage with consequent protection of the kidney function within the first four years after transplantation. After the fourth year the kidney protective effect of alemtuzumab levelled off, however a continuous trend could still be observed. We believe that the cumulative damage of calcineurin inhibitors on kidney function overtime is responsible for this effect. To the best of our knowledge the current study is the first one to demonstrate significant differences in renal function between different induction regimens in a large cohort of patients.

One of the principal safety concerns for the use of alemtuzumab is infectious complications and the risk to develop malignancies. So far, none of the previous studies in solid organ recipients demonstrated a higher risk of infectious complications in patients receiving alemtuzumab [20–23, 28–34]. This is in line with our analysis, where we did not find a higher rate of infections in the alemtuzumab group within the first year after transplantation, despite the consistently low leukocyte counts. More intriguingly, it appears as if the first episode of severe infection following transplantation occurs significantly earlier in NI and ATG groups. In the Alemtuzumab group, the peak incidence of the first infection was observed after the first year. Since the immunosuppressive effect of this agent seems to be minimal after 12 months, we speculate that alemtuzumab is not exerting a direct effect on incidence of infections after the first year. However, further studies are necessary to elucidate this finding. Incidence of *Pseudomonas* colonization was higher in the ATG group, which could be related to the fact that most of these patients had a pre-transplant diagnosis of CF. Occurrence of CMV disease was comparable in all groups. Supporting this trend, in all groups, Non-CMV infections were the most important cause of death.

There are some major limitations in this study. First of all, it is based on a retrospective analysis, including patients from a single centre. Compositions of the groups are naturally influenced by changes in the institutional routine and by availability of specific treatment possibilities. In order to reduce this limitations, we performed a multivariable analysis adjusting for time-effect. Furthermore, an era effect cannot be excluded since alemtuzumab was the most recently introduced induction therapy. Thus, the results might partially reflect recent improvements of postoperative care and long-term follow-up and not the specific immunosuppression regimen. Another limitation of the present study is that patients were not routinely screened for donor specific antibody formation and similarly, diagnostic workup for antibody-mediated rejection became a clinical routine only in the last years. Therefore, this could not be examined in this retrospective study.

In conclusion, the data presented herein revealed significant advantages of alemtuzumab over conventional induction agents. Its use facilitated the reduction of CNI dose which led to improved kidney function and a low risk of infections. Based on these results, we currently plan a randomized prospective study to further reduce the cumulative dose of tacrolimus and to investigate the immunomodulatory potential of alemtuzumab.

## Supporting information

S1 Table Univariate analysis for mortality risk. (DOCX)

S2 Table Univariate analysis for CLAD risk. (DOCX)

S3 Table Univariate analysis for ACR risk. (DOCX)

S4 Table Univariate analysis for LB risk. (DOCX)

S5 Table Multivariable analysis for mortality risk and CLAD risk. (DOCX)

S6 Table Multivariable analysis for ACR risk and LB risk. (DOCX)

S7 Table Number of patient at each time point. (DOCX)

## Abbreviations

ACR: acute cellular rejection
ATG: Anti-thymocyte globulin
ATS: American Thoracic society
BAL: bronchoalveolar lavage
CF: Cystic fibrosis
CLAD: chronic lung allograft dysfunction
CMV: Cytomegalovirus
CNI: Calcineurin-inhibitors
ECLS: Extracorporeal life support
ECMO: Extracirculatory membrane oxygenator
eGFR: estimated glomerular filtration rate
ERS: European Respiratory Society
IL2RA: IL-2 Receptor Antagonist
iPAH: idiopathic Pulmonary arterial Hypertension
ISHLT: International Society of Heart and Lung Transplantation
LB: Lymphocytic Bronchiolitis
MMF: Mycophenolat mofetil
NI: No induction
NK: Natural killer cells
POD: postoperative day
PTLD: post-transplant lympho-proliferative disease
TBB: Transbronchial biopsy
