# Supplementary material for: Alemtuzumab induction combined with reduced maintenance immunosuppression is associated with improved outcomes after lung transplantation: A single centre experience
Source: PLoS One. 2019 Jan 15;14(1):e0210443. doi: 10.1371/journal.pone.0210443 (PMC6333331; doi:10.1371/journal.pone.0210443)
Supplement: S1 Table — (DOCX) [file pone.0210443.s001.docx]

Supplementary Table 1 - *Univariate analysis for mortality risk*

|  | | | HR | 95.0% CI | | *p-value* |
| --- | --- | --- | --- | --- | --- | --- |
|  |  |  |  | Lower | Upper |  |
| Median age < 52 | | | 0.697 0.490 0.992 0.045 | | | |
| Type of Tx | | DLuTx | 0.599 0.313 1.149 0.123 | | | |
| Male sex | | | 1.293 0.911 1.835 0.151 | | | |
| Diagnosis | COPD | | 0.132  1.205 0.774 1.875 0.409  0.891 0.407 1.948 0.772  0.737 0.425 1.279 0.278  1.787 1.014 3.148 0.044 | | | |
|  | Fibrosis | |  |  |  |  |
|  | PH | |  |  |  |  |
|  | CF | |  |  |  |  |
|  | Others | |  |  |  |  |
| Induction therapy | No Induction | | 0.001  0.538 0.307 0.943 0.030  0.505 0.343 0.744 0.001 | | | |
|  | ATG | |  |  |  |  |
|  | Alemtuzumab | |  |  |  |  |
| Year of Tx | 2007 | | 0.024  0.963 0.509 1.821 0.908  0.429 0.204 0.903 0.026  0.769 0.402 1.474 0.429  0.453 0.213 0.964 0.040  0.869 0.431 1.751 0.694  0.317 0.132 0.759 0.010  0.573 0.257 1.275 0.173 | | | |
|  | 2008 | |  |  |  |  |
|  | 2009 | |  |  |  |  |
|  | 2010 | |  |  |  |  |
|  | 2011 | |  |  |  |  |
|  | 2012 | |  |  |  |  |
|  | 2013 | |  |  |  |  |
|  | 2014 | |  |  |  |  |
| LAS<50 | | | 0.554 0.355 0.864 0.009 | | | |
| Pre-Tx intubation | | | 1.546 0.927 2.580 0.095 | | | |
| Pre-Tx ECLS bridge | | | 1.601 0.883 2.903 0.121 | | | |
| CMV  risk | D-/R- | | 0.678  0.811 0.415 1.584 0.539  0.922 0.521 1.633 0.781  1.128 0.611 2.085 0.700 | | | |
|  | D+/R- | |  |  |  |  |
|  | D+/R+ | |  |  |  |  |
|  | D-/R+ | |  |  |  |  |
