# Supplementary material for: Alemtuzumab induction combined with reduced maintenance immunosuppression is associated with improved outcomes after lung transplantation: A single centre experience
Source: PLoS One. 2019 Jan 15;14(1):e0210443. doi: 10.1371/journal.pone.0210443 (PMC6333331; doi:10.1371/journal.pone.0210443)
Supplement: S2 Table — (DOCX) [file pone.0210443.s002.docx]

Supplementary Table 2 - *Univariate analysis for CLAD risk*

|  | | | HR | 95.0% CI | | *p-value* |
| --- | --- | --- | --- | --- | --- | --- |
|  |  |  |  | Lower | Upper |  |
| Median age < 52 | | | 0.929 | 0.636 | 1.358 | 0.704 |
| Type of Tx | | DLuTx | 0.933 | 0.426 | 2.041 | 0.861 |
| Male sex | | | 1.689 | 1.145 | 2.492 | 0.008 |
| Diagnosis | COPD | |  |  |  | 0.191 |
|  | Fibrosis | | 1.357 | 0.846 | 2.177 | 0.206 |
|  | PH | | 0.887 | 0.380 | 2.069 | 0.782 |
|  | CF | | 0.606 | 0.330 | 1.113 | 0.106 |
|  | Others | | 1.255 | 0.618 | 2.548 | 0.530 |
| Induction therapy | No Induction | |  |  |  | 0.000 |
|  | ATG | | 0.322 | 0.164 | 0.631 | 0.001 |
|  | Alemtuzumab | | 0.508 | 0.332 | 0.775 | 0.002 |
| Year of Tx | 2007 | |  |  |  | 0.439 |
|  | 2008 | | 0.846 | 0.234 | 3.061 | 0.799 |
|  | 2009 | | 0.929 | 0.289 | 2.984 | 0.901 |
|  | 2010 | | 0.782 | 0.243 | 2.514 | 0.680 |
|  | 2011 | | 0.903 | 0.287 | 2.836 | 0.861 |
|  | 2012 | | 0.946 | 0.302 | 2.960 | 0.924 |
|  | 2013 | | 1.796 | 0.583 | 5.534 | 0.308 |
|  | 2014 | | 1.475 | 0.474 | 4.593 | 0.502 |
| LAS<50 | | | .595 | .354 | 1.000 | 0.50 |
| Pre-Tx intubation | | | 0.612 | 0.348 | 1.075 | 0.088 |
| Pre-Tx ECLS bridge | | | 1.914 | 0.992 | 3.691 | 0.053 |
| CMV risk | D-/R- | |  |  |  | 0.280 |
|  | D+/R- | | 0.793 | 0.364 | 1.726 | 0.558 |
|  | D+/R+ | | 1.158 | 0.604 | 2.223 | 0.658 |
|  | D-/R+ | | 1.463 | 0.725 | 2.952 | 0.289 |
