# Supplementary material for: Alemtuzumab induction combined with reduced maintenance immunosuppression is associated with improved outcomes after lung transplantation: A single centre experience
Source: PLoS One. 2019 Jan 15;14(1):e0210443. doi: 10.1371/journal.pone.0210443 (PMC6333331; doi:10.1371/journal.pone.0210443)
Supplement: S3 Table — (DOCX) [file pone.0210443.s003.docx]

Supplementary Table 3 - *Univariate analysis for ACR risk*

|  | | | HR | 95.0% CI | | *p-value* |
| --- | --- | --- | --- | --- | --- | --- |
|  |  |  |  | Lower | Upper |  |
| Median age < 52 | | | 1.313 | 0.704 | 2.447 | 0.392 |
| Type of Tx | | DLuTx | .371 | .132 | 1.042 | .060 |
| Male sex | | | 0.813 | 0.437 | 1.512 | 0.513 |
| Diagnosis | COPD | |  |  |  | 0.145 |
|  | Fibrosis | | 0.433 | 0.150 | 1.251 | 0.122 |
|  | PH | | 1.745 | 0.663 | 4.590 | 0.259 |
|  | CF | | 0.481 | 0.166 | 1.392 | 0.177 |
|  | Others | | 1.400 | 0.484 | 4.051 | 0.535 |
| Induction therapy | No Induction | |  |  |  | 0.000 |
|  | ATG | | 0.412 | 0.145 | 1.171 | 0.096 |
|  | Alemtuzumab | | 0.150 | 0.065 | 0.341 | 0.000 |
| Year of Tx | 2007 | |  |  |  | 0.000 |
|  | 2008 | | 0.303 | 0.110 | 0.836 | 0.021 |
|  | 2009 | | 0.043 | 0.006 | 0.339 | 0.003 |
|  | 2010 | | 0.295 | 0.116 | 0.748 | 0.010 |
|  | 2011 | | 0.203 | 0.073 | 0.559 | 0.002 |
|  | 2012 | | 0.165 | 0.052 | 0.527 | 0.002 |
|  | 2013 | | 0.086 | 0.024 | 0.315 | 0.000 |
|  | 2014 | | 0.068 | 0.015 | 0.312 | 0.001 |
| LAS<50 | | | 0.948 | 0.371 | 2.419 | 0.910 |
| Pre-Tx intubation | | | 2.013 | 0.845 | 4.796 | 0.114 |
| Pre-Tx ECLS bridge | | | 2.013 | 0.845 | 4.796 | 0.114 |
| CMV risk | D-/R- | |  |  |  | .796 |
|  | D+/R- | | 1.339 | .412 | 4.349 | .627 |
|  | D+/R+ | | 1.179 | .401 | 3.467 | .764 |
|  | D-/R+ | | .829 | .243 | 2.832 | .765 |
