# Supplementary material for: Alemtuzumab induction combined with reduced maintenance immunosuppression is associated with improved outcomes after lung transplantation: A single centre experience
Source: PLoS One. 2019 Jan 15;14(1):e0210443. doi: 10.1371/journal.pone.0210443 (PMC6333331; doi:10.1371/journal.pone.0210443)
Supplement: S4 Table — (DOCX) [file pone.0210443.s004.docx]

Supplementary Table 4 - *Univariate analysis for LB risk*

|  | | | HR | 95.0% CI | | *p-value* |
| --- | --- | --- | --- | --- | --- | --- |
|  |  |  |  | Lower | Upper |  |
| Median age < 52 | | | 1.920 1.163 3.171 0.011 | | | |
| Type of Tx | | DLuTx | 0.970 0.304 3.089 0.958 | | | |
| Male sex | | | 1.077 0.664 1.748 0.763 | | | |
| Diagnosis | COPD | | 0.185  1.899 1.056 3.416 0.032  0.541 0.129 2.275 0.402  1.398 0.721 2.709 0.321  1.358 0.523 3.529 0.530 | | | |
|  | Fibrosis | |  |  |  |  |
|  | PH | |  |  |  |  |
|  | CF | |  |  |  |  |
|  | Others | |  |  |  |  |
| Induction therapy | No Induction | | 0.000  0.736 0.365 1.483 0.391  0.311 0.177 0.545 0.000 | | | |
|  | ATG | |  |  |  |  |
|  | Alemtuzumab | |  |  |  |  |
| Year of Tx | 2007 | | 0.000  0.761 0.363 1.595 0.470  0.315 0.132 0.750 0.009  0.223 0.091 0.546 0.001  0.184 0.072 0.469 0.000  0.180 0.063 0.513 0.001  0.189 0.074 0.484 0.001  0.032 0.004 0.245 0.001 | | | |
|  | 2008 | |  |  |  |  |
|  | 2009 | |  |  |  |  |
|  | 2010 | |  |  |  |  |
|  | 2011 | |  |  |  |  |
|  | 2012 | |  |  |  |  |
|  | 2013 | |  |  |  |  |
|  | 2014 | |  |  |  |  |
| LAS<50 | | | 0.561 0.300 1.050 0.071 | | | |
| Pre-Tx intubation | | | 2.395 1.253 4.577 0.008 | | | |
| Pre-Tx ECLS bridge | | | 1.634 0.706 3.784 0.252 | | | |
| CMV  risk | D-/R- | | 0.334  0.434 0.171 1.101 0.079  0.789 0.389 1.601 0.512  0.684 0.304 1.540 0.359 | | | |
|  | D+/R- | |  |  |  |  |
|  | D+/R+ | |  |  |  |  |
|  | D-/R+ | |  |  |  |  |
