# Supplementary material for: Alemtuzumab induction combined with reduced maintenance immunosuppression is associated with improved outcomes after lung transplantation: A single centre experience
Source: PLoS One. 2019 Jan 15;14(1):e0210443. doi: 10.1371/journal.pone.0210443 (PMC6333331; doi:10.1371/journal.pone.0210443)
Supplement: S5 Table — (DOCX) [file pone.0210443.s005.docx]

Supplementary Table 5 - *Multivariable analysis mortality risk*

|  | | HR | 95.0% CI | | *p-value* |
| --- | --- | --- | --- | --- | --- |
|  |  |  | Lower | Upper |  |
| Age < median (52y) | | 0.668 | .467 | .956 | .027 |
| Year of Tx | 2007 |  |  |  | 0.052 |
|  | 2008 | 1.239 | 0.624 | 2.460 | 0.540 |
|  | 2009 | **0.451** | **0.209** | **0.972** | **0.042** |
|  | 2010 | 0.881 | 0.446 | 1.743 | 0.716 |
|  | 2011 | 0.522 | 0.238 | 1.149 | 0.106 |
|  | 2012 | 0.797 | 0.391 | 1.623 | 0.531 |
|  | 2013 | 0.430 | 0.166 | 1.119 | 0.084 |
|  | 2014 | 0.788 | 0.313 | 1.986 | 0.614 |
| LAS > 50 | | 0.574 | 0.360 | 0.917 | **.020** |
| Induction therapy | No induction |  |  |  | 0.014 |
|  | ATG | **0.464** | **0.252** | **0.854** | **0.014** |
|  | Alemtuzumab | **0.600** | **0.374** | **0.961** | **0.033** |
| *Multivariable analysis for*  *CLAD risk* | |  | | | |
| Male sex | | 1.485 | 0.994 | 2.218 | 0.054 |
| LAS > 50 | | 0.638 | 0.370 | 1.100 | 0.106 |
| Year of Tx | 2007 |  |  |  | 0.379 |
|  | 2008 | 1.481 | 0.608 | 3.606 | 0.388 |
|  | 2009 | 0.890 | 0.352 | 2.251 | 0.805 |
|  | 2010 | 1.236 | 0.500 | 3.053 | 0.646 |
|  | 2011 | 1.283 | 0.504 | 3.265 | 0.601 |
|  | 2012 | 1.854 | 0.726 | 4.735 | 0.197 |
|  | 2013 | 2.510 | 0.884 | 7.127 | 0.084 |
|  | 2014 | 1.831 | 0.461 | 7.268 | 0.390 |
| Induction therapy | No induction |  |  |  | 0.001 |
|  | ATG | **0.351** | **0.171** | **0.722** | **0.004** |
|  | Alemtuzumab | **0.459** | **0.280** | **0.751** | **0.002** |
