# Supplementary material for: Alemtuzumab induction combined with reduced maintenance immunosuppression is associated with improved outcomes after lung transplantation: A single centre experience
Source: PLoS One. 2019 Jan 15;14(1):e0210443. doi: 10.1371/journal.pone.0210443 (PMC6333331; doi:10.1371/journal.pone.0210443)
Supplement: S6 Table — (DOCX) [file pone.0210443.s006.docx]

Supplementary Table 6 - *Multivariable analysis for ACR risk (≥A2) and LB risk*

|  | | | HR | 95.0% CI | | *p-value* |
| --- | --- | --- | --- | --- | --- | --- |
|  |  |  |  | Lower | Upper |  |
| Induction therapy | No Induction | |  |  |  | **.001** |
|  | ATG | | **.347** | **.113** | **1.065** | **.064** |
|  | Alemtuzumab | | **.168** | **.060** | **.474** | **.001** |
| Year of Tx | 2007 | |  |  |  | .068 |
|  | 2008 | | .581 | .198 | 1.707 | .323 |
|  | 2009 | | **.061** | **.008** | **.486** | **.008** |
|  | 2010 | | .476 | .182 | 1.243 | .130 |
|  | 2011 | | .373 | .132 | 1.058 | .064 |
|  | 2012 | | **.199** | **.062** | **.643** | **.007** |
|  | 2013 | | .313 | .074 | 1.329 | .115 |
|  | 2014 | | .330 | .055 | 1.991 | .227 |
| LAS<50 |  | | 1.122 | .425 | 2.963 | .816 |
|  | | |  | | | |
| Median age < 52 | | | **2.123** | **1.268** | **3.553** | **.004** |
| Pre-Tx intubation | | | 1.242 | .458 | 3.371 | .670 |
| Induction therapy | | No Induction |  |  |  | **.023** |
|  |  | ATG | **.427** | **.202** | **.905** | **.026** |
|  |  | Alemtuzumab | **.480** | **.243** | **.948** | **.035** |
| Year of Tx | | 2007 |  |  |  | .000 |
|  |  | 2008 | 1.229 | .554 | 2.728 | .611 |
|  |  | 2009 | **.321** | **.132** | **.783** | **.013** |
|  |  | 2010 | **.225** | **.089** | **.568** | **.002** |
|  |  | 2011 | **.238** | **.089** | **.633** | **.004** |
|  |  | 2012 | **.201** | **.069** | **.584** | **.003** |
|  |  | 2013 | **.322** | **.109** | **.950** | **.040** |
|  |  | 2014 | **.053** | **.006** | **.449** | **.007** |
| LAS<50 | | | 1.360 | .523 | 3.536 | .528 |
