# Supplementary material for: Alemtuzumab induction combined with reduced maintenance immunosuppression is associated with improved outcomes after lung transplantation: A single centre experience
Source: PLoS One. 2019 Jan 15;14(1):e0210443. doi: 10.1371/journal.pone.0210443 (PMC6333331; doi:10.1371/journal.pone.0210443)
Supplement: S7 Table — (DOCX) [file pone.0210443.s007.docx]

Supplementary Table 7 - *Number of patients per time point*

|  | Induction therapy | | |
| --- | --- | --- | --- |
|  | No (n=165) | ATG (n=50) | Alemtuzumab (n=231) |
| Before Tx | 153 | 49 | 229 |
| 3 months | 144 | 47 | 231 |
| 6 months | 135 | 46 | 209 |
| 12 months | 130 | 44 | 197 |
| 24 months | 121 | 40 | 141 |
| 3 years | 98 | 39 | 82 |
| ≥ 4 years | 98 | 38 | 82 |
